# Supplementary material for: The long lives of primates and the ‘invariant rate of ageing’ hypothesis
Source: Nat Commun. 2021 Jun 16;12:3666. doi: 10.1038/s41467-021-23894-3 (PMC8209124; doi:10.1038/s41467-021-23894-3)
Supplement: Supplementary file 1 — Supplementary Information [file 41467_2021_23894_MOESM1_ESM.pdf]

Supplementary Information

The long lives of primates and the 'invariant rate of ageing' hypothesis

Fernando Colchero*, José Manuel Aburto, Elizabeth A. Archie, Christophe Boesch, Thomas Breuer, Fernando A. Campos, Anthony Collins, Dalia A. Conde, Marina Cords, Catherine Crockford, Melissa Emery Thompson, Linda M. Fedigan, Claudia Fichtel, Milou Groenenberg, Catherine Hobaiter, Peter M. Kappeler, Richard R. Lawler, Rebecca J. Lewis, Zarin P. Machanda, Marie L. Manguette, Martin N. Muller, Craig Packer, Richard J. Parnell, Susan Perry, Anne E. Pusey, Martha M. Robbins, Robert M. Seyfarth, Joan B. Silk, Johanna Staerk, Tara S. Stoinski, Emma J. Stokes, Karen B. Strier, Shirley C. Strum, Jenny Tung, Francisco Villavicencio, Roman M. Wittig, Richard W. Wrangham, Klaus Zuberbühler, James W. Vaupel, Susan C. Alberts*

*Corresponding authors: colchero@imada.sdu.dk, alberts@duke.edu

This PDF file includes:

Supplementary Figures 1 to 6
Supplementary Tables 1 to 4

Supplementary Figure 1

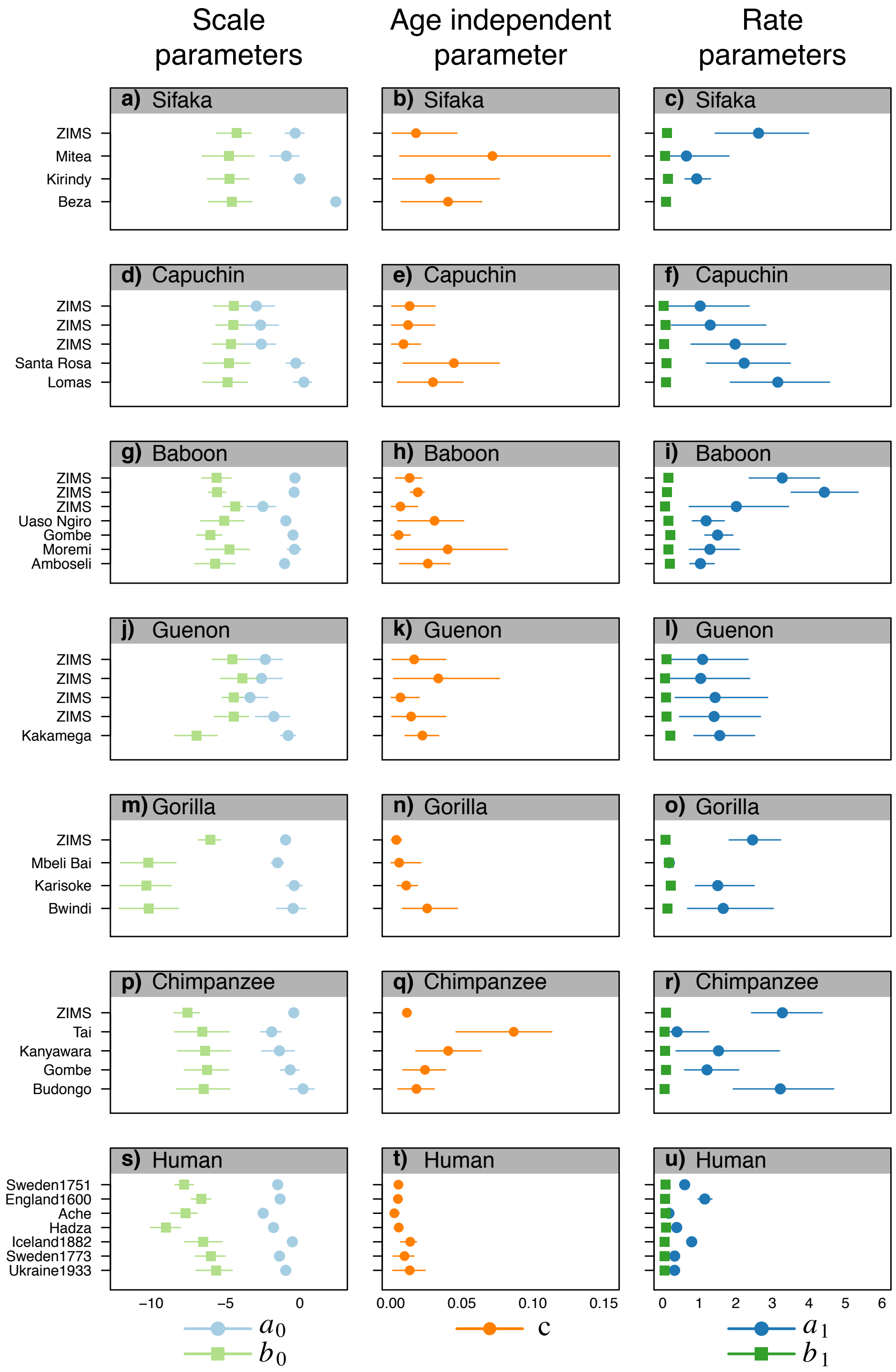

Supplementary Figure 1. Population-specific estimates of Siler mortality parameters (round or square points) and their 95% credible intervals for females. The parameters were organized in three categories (Columns of panels): 1) scale parameters (a_0, b_0); 2) age independent parameter (c); and 3) rate parameters (a_1, b_1). Each panel row corresponds to a genus: **a-c** Sifaka; **d-f** capuchin; **g-i** baboon; **j-l** guenon; **m-o** gorilla; **p-r** chimpanzee; **s-u** human.

Supplementary Figure 2

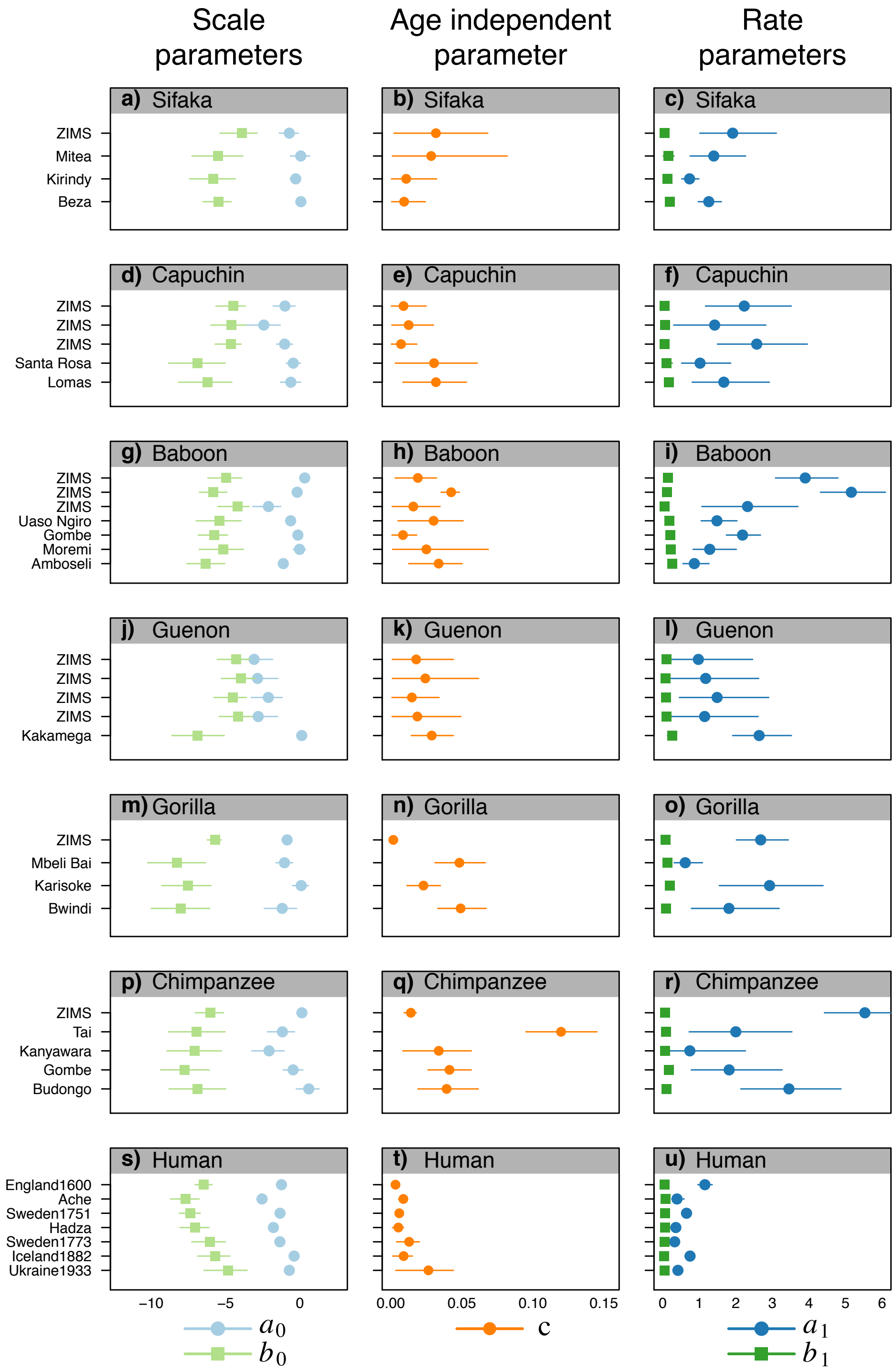

Supplementary Figure 2. Population-specific estimates of Siler mortality parameters (round or square points) and their 95% credible intervals for males. The parameters were organized in three categories (Columns of panels): 1) scale parameters (a_0, b_0); 2) age independent parameter (c); and 3) rate parameters (a_1, b_1). Each panel row corresponds to a genus: **a-c** Sifaka; **d-f** capuchin; **g-i** baboon; **j-l** guenon; **m-o** gorilla; **p-r** chimpanzee; **s-u** human.

Supplementary **Figure 3.**

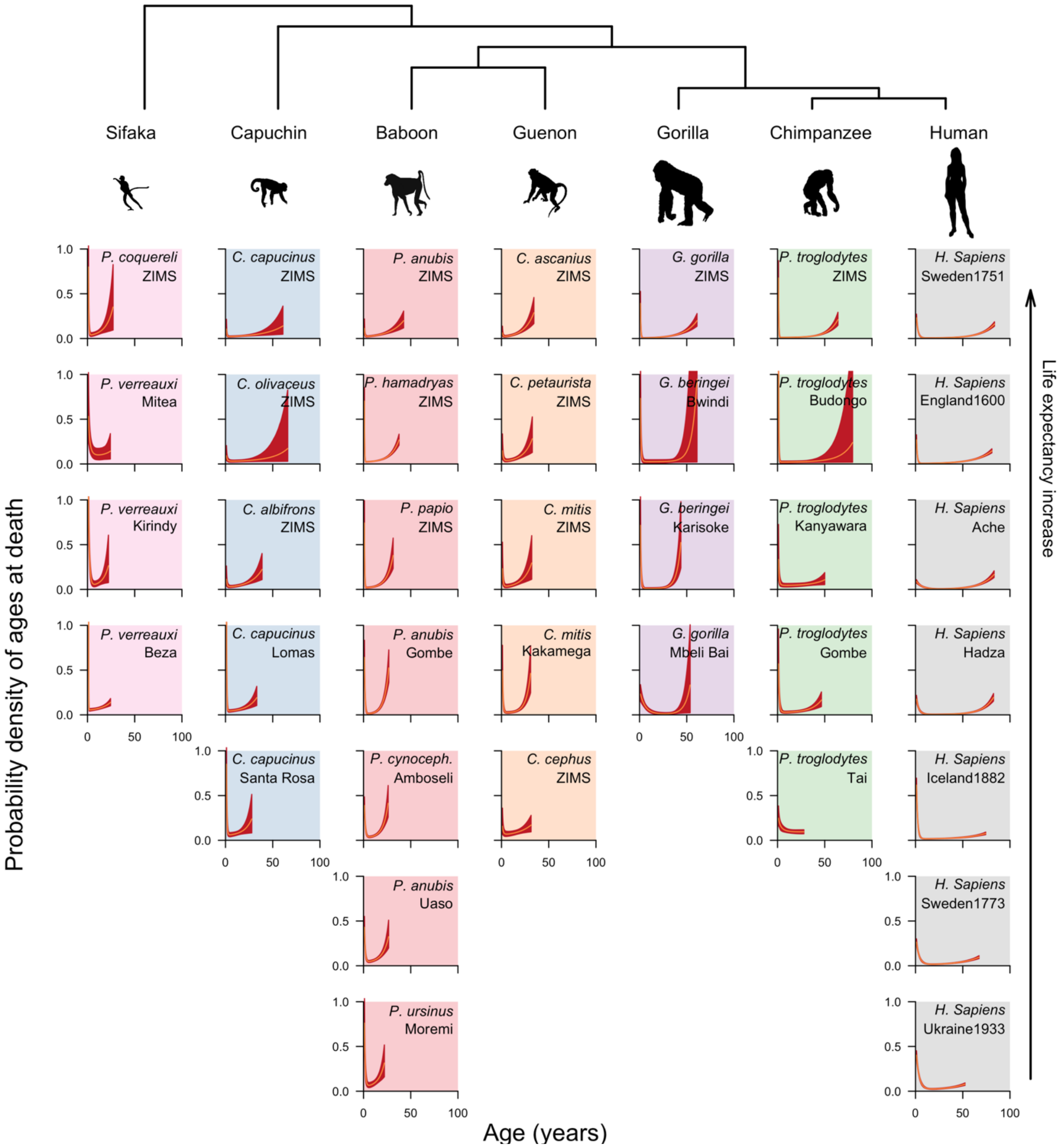

Supplementary Figure 3. Estimated age-specific mortality (i.e. hazard rates) for females in each population studied. For each genus, the direction of increase in life expectancy is from bottom to top, thus the population with lowest life expectancy appears at the bottom, while the one with highest life expectancy is at the top. The red polygons surrounding the orange average lines calculated from the posterior densities of the parameters denote the 95% credible intervals. All mortalities stop when the survival is $S(x) = 0.05$. For display purposes, we only included seven of the nine human populations. The primate silhouettes were obtained from http://phylopic.org/. Credits: sifaka by Terpsichores (Creative Commons Attribution-ShareAlike 3.0 Unported license), Capuchin by Sarah Werning (Creative Commons Attribution 3.0 Unported license), baboon by Owen Jones (Public Domain Dedication 1.0 license), guenon unknown author (Public Domain Mark 1.0 license), gorilla unknown author (Public Domain Mark 1.0 license), chimpanzee by T. Michael Keesey and Tony Hisgett (Creative Commons Attribution 3.0 Unported license), human by unknown author (Public Domain Mark 1.0 license)

Supplementary **Figure 4.**

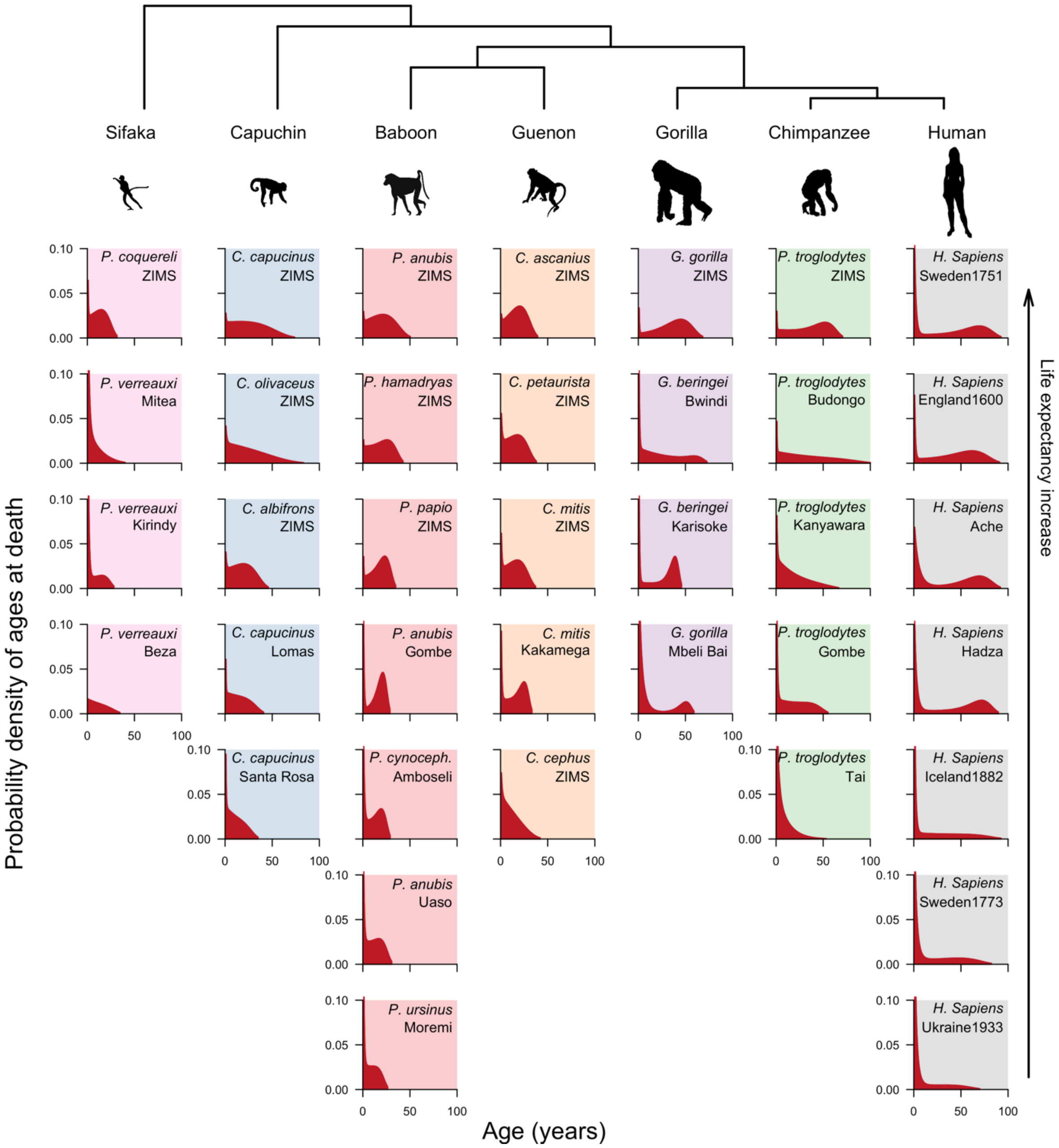

Supplementary Figure 4. Probability density of ages at death for females in the populations studied. For each genus, the direction of increase in life expectancy is from bottom to top, thus the population with lowest life expectancy appears at the bottom, while the one with highest life expectancy is at the top. For display purposes, we only included seven of the nine human populations. The primate silhouettes were obtained from http://phylopic.org/. Credits: sifaka by Terpsichores (Creative Commons Attribution-ShareAlike 3.0 Unported license), Capuchin by Sarah Werning (Creative Commons Attribution 3.0 Unported license), baboon by Owen Jones (Public Domain Dedication 1.0 license), guenon unknown author (Public Domain Mark 1.0 license), gorilla unknown author (Public Domain Mark 1.0 license), chimpanzee by T. Michael Keesey and Tony Hisgett (Creative Commons Attribution 3.0 Unported license), human by unknown author (Public Domain Mark 1.0 license).

Supplementary **Figure 5.**

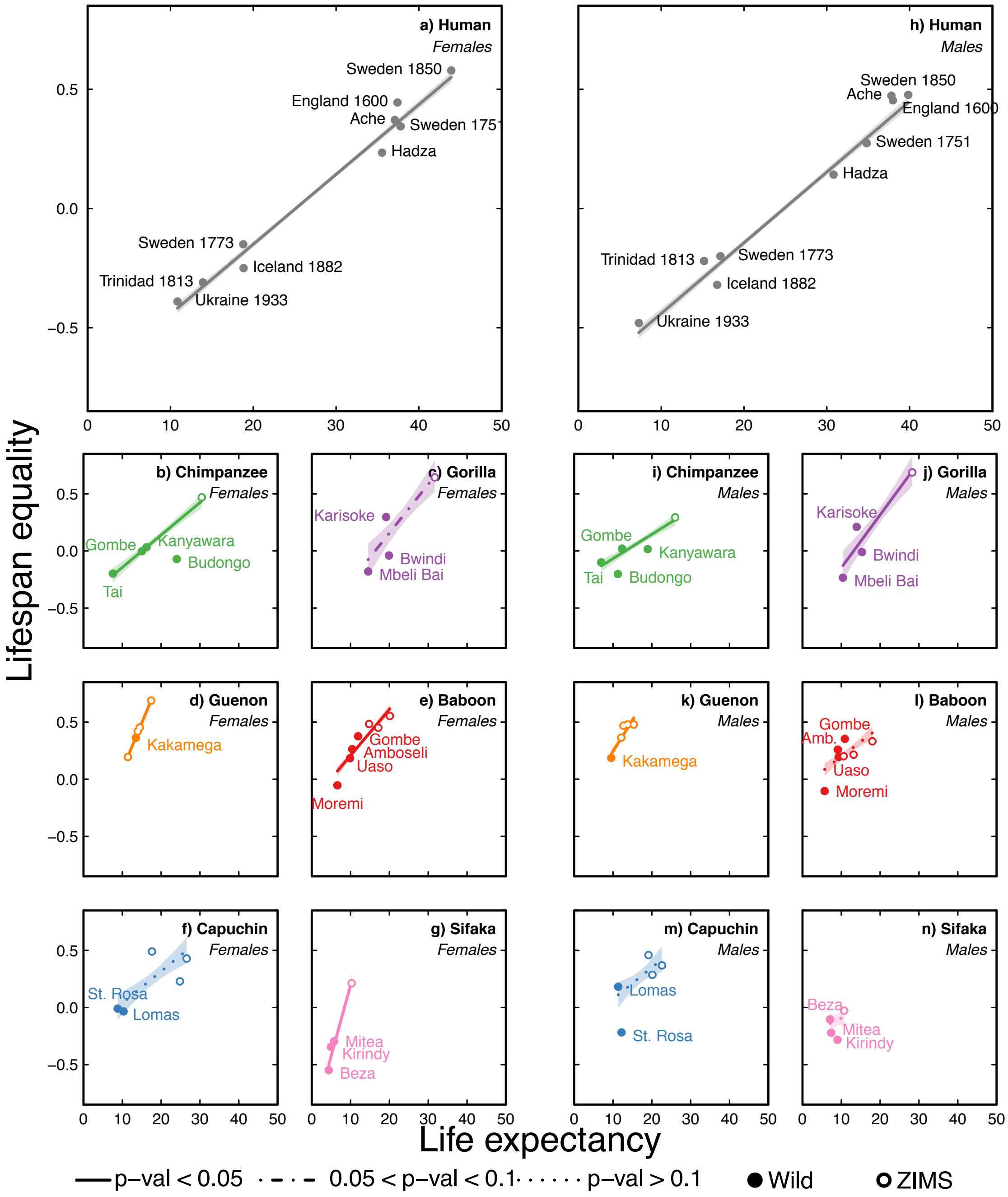

Supplementary Figure 5. Scatterplots of life expectancy and lifespan equality for all genera studied. The lines show the weighted regression lines and the open circles show the populations from the ZIMS database. The central lines are the predicted fitted values of the regression and the type of line (e.g. continuous, dashed, or dotted) depicts three levels for the *p*-values of the slopes (how significantly different from 0 they are, two-sided *t*-test, H_0: $\beta_1 = 0$, Supplementary Table 4), while the shaded polygons show the 95% confidence intervals of the regressions. Panels **a-g** correspond to the female data, and panels **h-n** to the male data.

Supplementary Figure 6

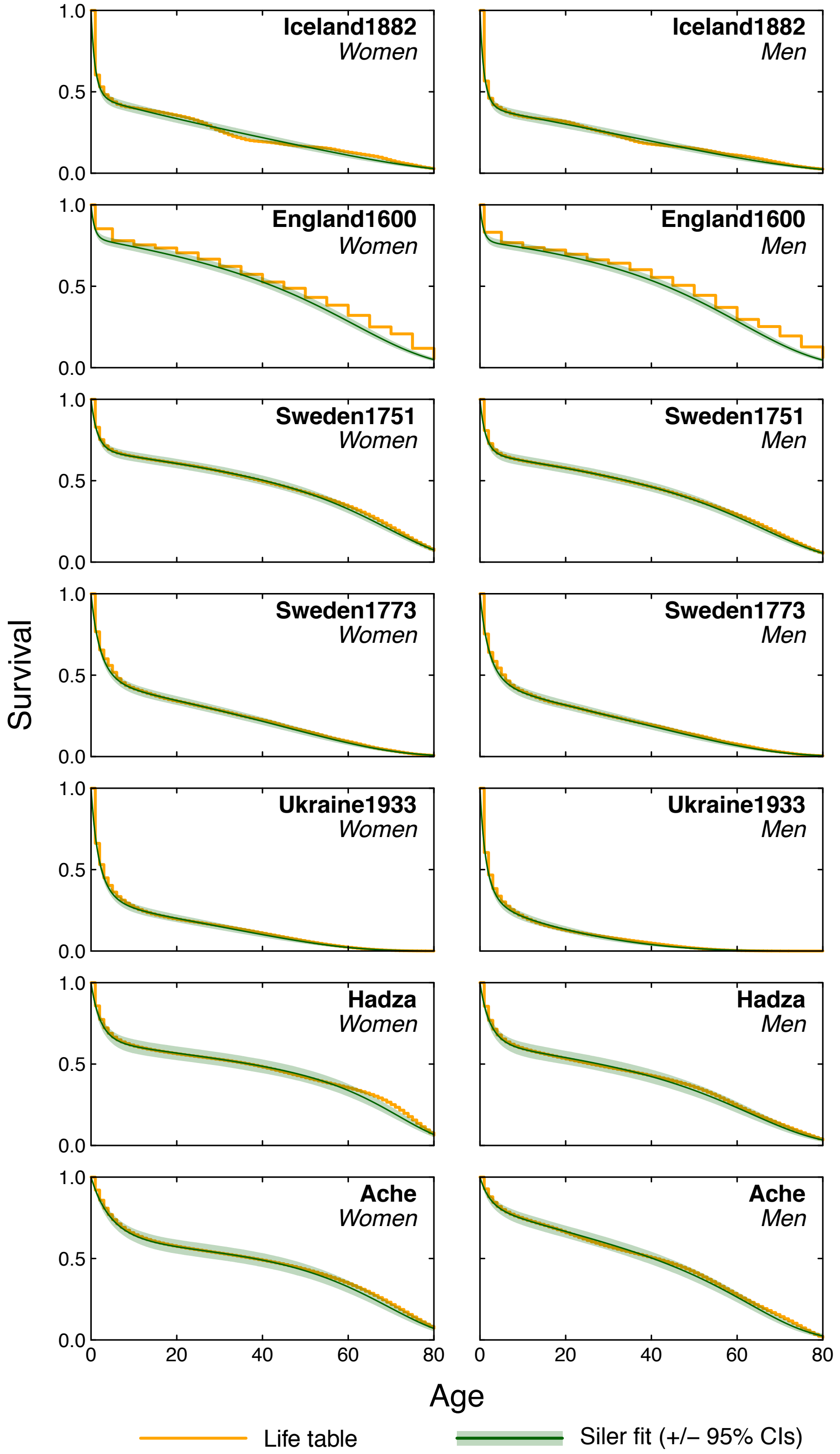

Supplementary Figure 6. Fit of the Siler model to the human life table data. For display purposes we only show seven of the nine human populations.

Supplementary Table 1. Weighted least squares regression between life expectancy and lifespan equality for each genus and sex. The *p*-value corresponds to the slope coefficient by means of a two-sided *t*-test (H_0: $\beta_1 = 0$). Those in boldface correspond to significant slopes (i.e. *p*-value < 0.05).

Sex	Genera	Intercept	Slope	SE(slope)	*p*-value
Females	Human	-0.736	0.0293	0.0012	**< 0.001**
	Chimpanzee	-0.428	0.0284	0.0045	**0.008**
	Gorilla	-0.696	0.0426	0.0113	0.063
	Guenon	-0.739	0.0820	0.0018	**< 0.001**
	Baboon	-0.196	0.0403	0.0078	**0.004**
	Capuchin	-0.240	0.0278	0.0119	0.102
	Sifaka	-1.048	0.1244	0.0189	**0.022**
Males	Human	-0.737	0.0296	0.0014	**< 0.001**
	Chimpanzee	-0.279	0.0212	0.0047	**0.020**
	Gorilla	-0.611	0.0463	0.0101	**0.045**
	Guenon	-0.296	0.0541	0.0134	**0.027**
	Baboon	-0.063	0.0261	0.0154	0.152
	Capuchin	-0.202	0.0276	0.0171	0.204
	Sifaka	-0.256	0.0162	0.0363	0.698

Supplementary Table 2. Sensitivity of life expectancy and lifespan equality to each Siler mortality parameter for the seven genera. These sensitivities were calculated at the mid-points of the regression lines for each genus.

Life expectancy			**Parameters**		
**Common**	a_0	a_1	c	b_0	b_1
Sifaka	-1.193	0.815	-61.952	-0.517	-6859.547
Capuchin	-7.063	4.903	-273.40	-6.253	-1500.227
Baboon	-4.454	2.826	-146.365	-3.994	-449.163
Guenon	-4.133	2.186	-162.517	-3.434	-354.234
Gorilla	-9.052	5.045	-459.384	-7.846	-2920.622
Chimpanzee	-9.847	6.783	-354.661	-6.853	-2425.216
Human	-12.926	8.930	-754.896	-9.316	-7985.996
Lifespan equality			**Parameters**		
**Common**	a_0	a_1	c	b_0	b_1
Sifaka	-0.129	0.080	0.184	0.002	848.219
Capuchin	-0.505	0.345	-13.35	-0.096	19.305
Baboon	-0.460	0.286	-10.508	-0.071	9.418
Guenon	-0.439	0.229	-12.508	-0.046	7.571
Gorilla	-0.517	0.286	-18.375	-0.099	25.756
Chimpanzee	-0.570	0.387	-13.936	-0.087	25.009
Human	-0.498	0.340	-18.27	-0.057	85.455

Supplementary Table 3. Level of collinearity between the genus regression lines and the sensitivity vectors to each mortality parameter for the seven genera. Collinearities were calculated with respect to the sensitivities in Supplementary Table 1.

Common	a_0	a_1	c	b_0	b_1
Sifaka	0.6293	0.5950	0.1767	0.1863	0.9638
Capuchin	0.9615	0.9623	0.9894	0.9468	0.4112
Baboon	0.9980	0.9998	0.9893	0.8122	0.3901
Guenon	0.9769	0.9422	0.9490	0.5296	0.1708
Gorilla	0.9963	0.9999	0.9967	0.8693	0.1282
Chimpanzee	0.9510	0.9526	0.9879	0.9284	0.3251
Human	0.9696	0.9704	0.9677	0.8574	0.2446

Supplementary Table 4. Relative amount of change for each mortality parameter along the genus lines for both sexes. The relative amount of change was calculated by means of the path integral described in the methods and Supplementary Methods sections.

Females					
**Common**	a_0	a_1	c	b_0	b_1
Sifaka	1.098	1.953	0.774	0.312	0.136
Capuchin	0.423	0.296	2.839	0.051	0.014
Baboon	0.574	0.775	4.374	0.078	0.029
Guenon	0.736	0.849	8.598	0.067	0.021
Gorilla	0.579	0.252	8.407	0.036	0.007
Chimpanzee	0.507	0.296	7.917	0.072	0.023
Human	1.236	0.168	2.235	0.032	0.005
Males					
**Common**	a_0	a_1	c	b_0	b_1
Sifaka	0.56	1.013	0.169	0.247	0.101
Capuchin	0.417	0.247	5.629	0.046	0.012
Baboon	0.519	0.535	3.082	0.087	0.035
Guenon	0.578	0.561	7.835	0.070	0.025
Gorilla	0.564	0.338	9.576	0.048	0.011
Chimpanzee	0.489	0.632	2.890	0.075	0.025
Human	8.993	0.153	4.022	0.041	0.007
