# Supplementary material for: The long lives of primates and the ‘invariant rate of ageing’ hypothesis
Source: Nat Commun. 2021 Jun 16;12:3666. doi: 10.1038/s41467-021-23894-3 (PMC8209124; doi:10.1038/s41467-021-23894-3)
Supplement: Supplementary file 3 — Description of Additional Supplementary Files [file 41467_2021_23894_MOESM3_ESM.pdf]

Description of Additional Supplementary Files

File Name: Supplementary Data 1
Description: Non-human primate populations analyzed for this study. The last four columns refer to N = total number of individuals, F = number of females, M = number of males, and U = number of individuals with undetermined sex.

File Name: Supplementary Data 2
Description: Siler parameter values sampled from the Bayesian survival analysis for both sexes on the 39 populations studied. The initial number corresponds to the mean of the parameter posterior density, and the numbers in parentheses are the 95% credible intervals.

File Name: Supplementary Data 3
Description: Female and male life expectancy and lifespan equality for the 39 populations of human and non-human primates.
