# Supplementary material for: The long lives of primates and the ‘invariant rate of ageing’ hypothesis
Source: Nat Commun. 2021 Jun 16;12:3666. doi: 10.1038/s41467-021-23894-3 (PMC8209124; doi:10.1038/s41467-021-23894-3)
Supplement: Supplementary file 7 — Reporting Summary [file 41467_2021_23894_MOESM7_ESM.pdf]

nature research

Corresponding author(s): Fernando Colchero and Susan Alberts

Last updated by author(s): May 17, 2021

Reporting Summary

Nature Research wishes to improve the reproducibility of the work that we publish. This form provides structure for consistency and transparency in reporting. For further information on Nature Research policies, see our Editorial Policies and the Editorial Policy Checklist.

Statistics

For all statistical analyses, confirm that the following items are present in the figure legend, table legend, main text, or Methods section.

n/a	Confirmed	
☐	☒	The exact sample size (*n*) for each experimental group/condition, given as a discrete number and unit of measurement
☐	☒	A statement on whether measurements were taken from distinct samples or whether the same sample was measured repeatedly
☐	☒	The statistical test(s) used AND whether they are one- or two-sided<br>*Only common tests should be described solely by name; describe more complex techniques in the Methods section.*
☐	☒	A description of all covariates tested
☐	☒	A description of any assumptions or corrections, such as tests of normality and adjustment for multiple comparisons
☐	☒	A full description of the statistical parameters including central tendency (e.g. means) or other basic estimates (e.g. regression coefficient) AND variation (e.g. standard deviation) or associated estimates of uncertainty (e.g. confidence intervals)
☐	☒	For null hypothesis testing, the test statistic (e.g. *F*, *t*, *r*) with confidence intervals, effect sizes, degrees of freedom and *P* value noted<br>*Give P values as exact values whenever suitable.*
☐	☒	For Bayesian analysis, information on the choice of priors and Markov chain Monte Carlo settings
☒	☐	For hierarchical and complex designs, identification of the appropriate level for tests and full reporting of outcomes
☒	☐	Estimates of effect sizes (e.g. Cohen's *d*, Pearson's *r*), indicating how they were calculated

Our web collection on statistics for biologists contains articles on many of the points above.

Software and code

Policy information about availability of computer code

Data collection	Data collection did not require any specific code. For all analyses, we generate all our code in the open source software R version 4.0.3 (2020-10-10)
Data analysis	The code showing an overview of the analyses used in the manuscript is available at https://github.com/fercol/ColcheroEtal2021NatComm (DOI: 10.5281/zenodo.4736892)

For manuscripts utilizing custom algorithms or software that are central to the research but not yet described in published literature, software must be made available to editors and reviewers. We strongly encourage code deposition in a community repository (e.g. GitHub). See the Nature Research guidelines for submitting code & software for further information.

Data

Policy information about availability of data

All manuscripts must include a data availability statement. This statement should provide the following information, where applicable:

- Accession codes, unique identifiers, or web links for publicly available datasets
- A list of figures that have associated raw data
- A description of any restrictions on data availability

Full datasets for survival analyses on wild populations and populations under human care of non-human primate species supporting the findings of this study were used under license for the current study and are not publicly available; specific requests for the access to the wild data should be addressed to the PIs of the data. Data of animals under human care are however available from Species360 (https://www.species360.org/) upon reasonable request. Summarized data underlying the analyses here and sufficient to calculate life tables and summary statistics such as life expectancy and lifespan equality are available in the Dryad data repository, URL: https://doi.org/10.5061/dryad.4b8gthtb4. Data from human populations was obtained from the Human Mortality Database (https://www.mortality.org/) and published sources. Source data are provided with this paper.

Field-specific reporting

Please select the one below that is the best fit for your research. If you are not sure, read the appropriate sections before making your selection.

☐ Life sciences ☐ Behavioural & social sciences ☒ Ecological, evolutionary & environmental sciences

For a reference copy of the document with all sections, see nature.com/documents/nr-reporting-summary-flat.pdf

Ecological, evolutionary & environmental sciences study design

All studies must disclose on these points even when the disclosure is negative.

Study description	We used existing life history data on primates and humans, stored in existing databases, to conduct analyses of mortality patterns. No new data were collected for this study. We ran parametric survival analyses based on the method proposed by Colchero et al (2016) and Barthold et al (2016) on 30 populations of non-human primates using the Siler mortality model. We also obtained life table information for 9 human populations. We then used the resulting age-specific survival to estimate life expectancy and lifespan equality as in Colchero et al (2016). We found that the linear relationship between these to variables we found among humans in Colchero et al (2016) was reproduced in other genera of non-human primates. To explain these regularities, we then carried out mathematical derivations and proofs for the sensitivities of life expectancy and lifespan equality to changes in mortality parameters, using the Siler parametric model as reference. We showed that changes in life expectancy and lifespan equality along the genus lines were mostly determined by changes in the mortality parameters that govern immature mortality and age-independent mortality, but that the parameters that determine adult, and particularly, senescence mortality (i.e. ageing rates), changed orders of magnitude less.
Research sample	We obtained 30 datasets for six genera of non-human primates: sifaka (Propithecus spp), gracile capuchin monkey (Cebus spp), guenon (Cercopithecus spp), baboon (Papio spp), gorilla (Gorilla spp), and chimpanzee (Pan troglodytes) (Supplementary Data 1). Of these, 17 datasets correspond to long-term projects in the wild, while 13 were contributed by the non-profit Species360 from the Zoological Information Management System (ZIMS)18, which is the most extensive database of life history information for animals under human care.
Sampling strategy	We used all available life history data from all populations. We did not carry out analyses to determine sample sizes. The sample sizes of the primate populations ranged from 146 to 4620 individuals. We accepted the smaller sample size based on the assessment by Colchero and Clark (2012) on a similar method.
Data collection	The data were recorded separately for each study over multiple decades, using individual registration and recording all observed births, deaths, and disappearances.
Timing and spatial scale	Each study had a separate start and stop date. We used all available data between the earliest and latest known individual record for each study. The temporal and spatial scales varied between studies, but the shortest study was of 12 years (Sifaka Mitea), and the longest study was of 54 years (Gombe chimpanzee). The average study duration was 34 years. The spatial span of each study varied as a function of the study groups' ranges.
Data exclusions	No data were excluded.
Reproducibility	No experiments were undertaken. The survival analyses and the numerical approximations to our mathematical derivations are fully reproducible.
Randomization	No allocation into experimental groups occurred.
Blinding	Life history data were collected on known individuals. Blinding was not appropriate for data collection.

Did the study involve field work? ☒ Yes ☐ No

Field work, collection and transport

Field conditions	Each of the studies had different field conditions. Reporting all of them is prohibitive but details of each study can be found in study-specific materials cited in the text.
Location	Data were collected in many countries around the world, including Botswana, Brazil, Costa Rica, Côte d'Ivoire, Kenya, Madagascar, Uganda, Republic of Congo, Rwanda, and Tanzania. The zoo data were similarly collected from zoos all over the world.
Access & import/export	No biological samples were collected for this analysis, making import and export unnecessary. Observational data were collected with the permission of the governments of Botswana, Brazil, Costa Rica, Côte d'Ivoire, Kenya, Madagascar, Uganda, Republic of Congo, Rwanda, and Tanzania
Disturbance	No disturbance of animals occurred during data collection.

Reporting for specific materials, systems and methods

We require information from authors about some types of materials, experimental systems and methods used in many studies. Here, indicate whether each material, system or method listed is relevant to your study. If you are not sure if a list item applies to your research, read the appropriate section before selecting a response.

Materials & experimental systems

n/a	Involved in the study
☒	☐ Antibodies
☒	☐ Eukaryotic cell lines
☒	☐ Palaeontology and archaeology
☐	☒ Animals and other organisms
☒	☐ Human research participants
☒	☐ Clinical data
☒	☐ Dual use research of concern

Methods

n/a	Involved in the study
☒	☐ ChIP-seq
☒	☐ Flow cytometry
☒	☐ MRI-based neuroimaging

Animals and other organisms

Policy information about studies involving animals; ARRIVE guidelines recommended for reporting animal research

Laboratory animals	No laboratory animals were used in the analysis. We obtained 13 datasets for primates in zoos representing six genera of non-human primates: sifaka (Propithecus spp), gracile capuchin monkey (Cebus spp), guenon (Cercopithecus spp), baboon (Papio spp), gorilla (Gorilla spp), and chimpanzee (Pan troglodytes) (Supplementary Data 1). These were contributed by the non-profit Species360 from the Zoological Information Management System (ZIMS).
Wild animals	Our dataset incorporates individual, longitudinal life history data from long-term field studies of wild primates into a synthetic database for comparative analyses. We obtained 17 datasets from wild primates representing six genera of non-human primates: sifaka (Propithecus spp), gracile capuchin monkey (Cebus spp), guenon (Cercopithecus spp), baboon (Papio spp), gorilla (Gorilla spp), and chimpanzee (Pan troglodytes) (Supplementary Data 1).
Field-collected samples	No biological samples were involved in this analysis.
Ethics oversight	All research on wild primates complied with ethical guidelines in the host countries: Botswana, Brazil, Costa Rica, Côte d'Ivoire, Kenya, Madagascar, Uganda, Republic of Congo, Rwanda, and Tanzania. In addition, each of the PIs obtained ethical clearance from the Animal Care and Use Committees of their institutions.

Note that full information on the approval of the study protocol must also be provided in the manuscript.
